# Supplementary material for: Network-driven differences in mobility and optimal transitions among automatable jobs
Source: R Soc Open Sci. 2019 Jul 3;6(7):182124. doi: 10.1098/rsos.182124 (PMC6689632; doi:10.1098/rsos.182124)
Supplement: Supplementary material [file rsos182124supp1.pdf]

Supplementary material from "Network-driven differences in mobility and optimal transitions among automatable jobs"

Jordan D. Dworkin[a]

[a]*Department of Biostatistics, Epidemiology, and Informatics, Perelman School of Medicine, University of Pennsylvania, Philadelphia, PA, USA*

Summary

The potential for widespread job automation has become an important topic of discussion in recent years, and it is thought that many American workers may need to learn new skills or transition to new jobs to maintain stable positions in the workforce. Because workers' existing skills may make such transitions more or less difficult, the likelihood of a given job being automated only tells part of the story. As such, this study utilizes network science and statistics to investigate the links between jobs that arise from their necessary skills, knowledge, and abilities. The resulting network structure is found to enhance the burden of automation within some sectors, while lessening the burden in others. Additionally, a model is proposed for quantifying expected benefit of specific job transitions. Its optimization reveals that the consideration of shared skills yields better transition recommendations than automatability and job growth alone. Finally the potential benefit of increasing individual skills is quantified, with respect to both job transitions and within-occupation skill redefinition. Broadly, this study presents a framework for measuring the links between jobs, and demonstrates the importance of these links for understanding the complex effects of automation.

May 13, 2019

Supplemental methods

Validation of similarity measure

The validity of the proposed measure of pairwise job similarity was tested by comparing a job's similarities to every other job to the rate at which workers transitioned between that job and every other job. Transition data was drawn from the Current Population Survey (CPS) [1], and jobs were matched between the O*Net job classifications and the CPS job classifications using the SOC to OCC crosswalk from the Bureau of Labor Statistics. After removing jobs that did not have a one-to-one match between the O*Net classification and the CPS classification, as well as jobs that had fewer than 100 total workers surveyed in the CPS sample, 277 jobs remained for validation.

For each of the 277 jobs, the Spearman correlation between its similarity values and its rate of transitions with the other 276 jobs. Transition rate was defined as the number of transitions between the job of interest, job i, and another job, job j, divided by the total number of workers surveyed who had reported working in job j at some point in the 2011-2016 sample. This ratio was used to account for the fact that jobs with many surveyed workers would have inflated transition counts. Thus the transition rate value represents the number of transitions between jobs i and j per worker in job j. Since correlations were conducted within each job i, these rates were not scaled by the number of workers in job i.

To determine the degree of correspondence between similarity values and transition rates, the weighted average of the 277 correlations was computed. This average was weighted by the total number of participants surveyed who reported working in job i at some point in the 2011-2016 sample. Thus the average upweights the correlations within jobs for which there was more transition data available. The weighted average of the similarity-transition correlations was found to be 0.43 (Fig. S1), suggesting that the proposed similarity measure tends to capture the propensity of workers to transition between job pairs. Figure S1 shows the relationship between within-job correlations and the number of workers surveyed from that job, as well as the distribution of all within-job correlation values. It additionally shows an example of the relationship between similarities and transition rates among *laborers and freight, stock, and material movers.*

Transition model

For the proposed transition model, the benefit of a transition from job i to job j can be quantified as the decrease in automatability from job i to job j, combined with binary markers that a worker 1) successfully completed retraining and 2) was able to find and be hired in job j. This benefit then takes the form:

$$b_{ij} = (a_j - a_i)\xi_{ij}\lambda_j,$$

$$\xi_{ij} \sim Bern(p^r_{ij}),$$

$$\lambda_j \sim Bern(p^h_j),$$

where ξ_{ij} is a Bernoulli random variable representing the successful completion of retraining from job i to job j, with probability p^r_{ij}, and λ_j is a Bernoulli random variable representing successfully being hired for job j, with probability p^h_j. In this study, the retraining probability was based on the similarity between job i and job j, where higher similarity yielded a higher probability of successful retraining. The successful hiring probability was based on the projected growth of job j, where

more open jobs yielded a higher probability of a successful hire. These probabilities were defined as:

$$p^r_{ij} = \frac{c_u - c_l}{max(S^i) - min(S^i)}(s_{ij} - min(S^i)) + c_l,$$

$$p^h_j = \frac{log(e^g_j)^\sigma}{max(log(e^g)^\sigma)},$$

where c_l and c_u are the lower and upper bounds of the retraining probability, to which the job similarities are rescaled, S^i is the set of similarities between job i and all other jobs, e is the set of all employment growth values, and σ tunes the degree of skewness in the hiring probabilities.

To optimize the expected benefit function, several values within these probabilities must be preselected. Specifically, c_l and c_u must be set, representing the probabilities of successful transition from a job to its most different and most similar jobs, respectively. For the primary analysis, $c_l = 0.25$ and $c_u = 0.75$ were used, and the effects of narrower and wider ranges are shown in Figure S1. The σ parameter must also be selected to determine the manner in which job growth translates to the probability of a trained worker successfully finding an open job j. For the primary analysis, σ was set at 1, and the effects of larger or smaller values are shown in Figure S2.

Given these values, for any given transition the expected benefit can be calculated as:

$$E(b_{ij}) = (a_j - a_i)p^r_{ij}p^h_j.$$

From this, the benefit of a set of transition recommendations for a worker in job i can be calculated. This measure is simply the benefit of the specific transition the worker chooses from the set, so for a given set of recommendations it is the sum over all recommended jobs of the product of 1) a binary marker that the worker selected that job for a transition and 2) the benefit of that transition. This overall benefit then takes the form:

$$b_i = \sum_{j \in T^i_n} I(\tau^i = j)b_{ij},$$

$$\tau^i \sim Mult(1, \pi^t_i),$$

where T^i_n is the set of jobs with the top n highest transition scores for job i, τ^i is a multinomial random variable representing the job selected for transition from job i, and π^t_i is the set of transition probabilities for jobs within T^i_n. These probabilities are given by the relative transition scores of each job in the set, with the form:

$$\begin{aligned}\pi^t_i &= \left(\pi^t_{i1}, \pi^t_{i2}, ..., \pi^t_{in}\right) \\ &= \left(\frac{t^i_1(w)}{\sum_{j \in T^i_n} t^i_j(w)}, \frac{t^i_2(w)}{\sum_{j \in T^i_n} t^i_j(w)}, ..., \frac{t^i_n(w)}{\sum_{j \in T^i_n} t^i_j(w)}\right).\end{aligned}$$

Then the expected benefit for job i arising from a particular set of transition scores is:

$$E(b_i) = \sum_{j \in T^i_n} \pi^t_{ij} E(b_{ij}).$$

For the calculation of $E(b_i)$, the choice of n within T_n^i must also be pre-selected. This value, representing the number of recommended transitions available to a worker in job i, was set at 20 for the primary analysis. The effects of lower and higher values of n are shown in Figure S3. The proposed model used to calculate the expected benefit of a set of transition recommendations for workers in a given job was implemented using an in-house R script. Optimization of this outcome over the space of w_a, w_g, and w_s was carried out using the Nelder-Mead method [2], where values were bounded such that $w_a + w_g + w_s = 1$.

Supplemental results

	20% (n = 138)		15% (n = 104)		10% (n = 70)		5% (n = 36)		1% (n = 8)	
Effect	t_{677}	p	t_{677}	p	t_{677}	p	t_{677}	p	t_{677}	p
Business - Scientific	5.62	< 0.001***	4.23	< 0.001**	2.98	< 0.01	1.52	> 0.05	2.87	< 0.01
Business - Medical	3.57	< 0.001*	2.95	< 0.01	1.70	> 0.05	0.78	> 0.05	-0.03	> 0.05
Business - Service	20.41	< 0.001***	18.59	< 0.001***	16.64	< 0.001***	14.48	< 0.001***	12.33	< 0.001***
Business - Industrial	43.79	< 0.001***	41.11	< 0.001***	37.52	< 0.001***	34.30	< 0.001***	31.41	< 0.001***
Scientific - Medical	-1.08	> 0.05	-0.58	> 0.05	-0.74	> 0.05	-0.46	> 0.05	-2.19	< 0.05
Scientific - Service	12.90	< 0.001***	12.45	< 0.001***	11.79	< 0.001***	11.11	< 0.001***	8.21	< 0.001***
Scientific - Industrial	29.33	< 0.001***	28.49	< 0.001***	26.81	< 0.001***	25.61	< 0.001***	22.10	< 0.001***
Medical - Service	12.61	< 0.001***	11.73	< 0.001***	11.29	< 0.001***	10.41	< 0.001***	9.46	< 0.001***
Medical - Industrial	26.67	< 0.001***	25.41	< 0.001***	24.12	< 0.001***	22.78	< 0.001***	21.56	< 0.001***
Service - Industrial	15.20	< 0.001***	14.87	< 0.001***	13.90	< 0.001***	13.47	< 0.001***	13.29	< 0.001***
Automatability	12.04	< 0.001***	11.89	< 0.001***	12.01	< 0.001***	11.68	< 0.001***	9.82	< 0.001***

Table S1: **The effect of the pre-selected neighborhood size on estimated mobility effects**. Rows represent effects reported for the upwards mobility measure, columns represent their statistic values and statistical significance for different choices of neighborhood size. First ten row represent mobility differences between job sectors, accounting for automatability of each job. Last row represents the association between automatability and mobility, accounting for job sector. P-values in the table are not corrected for multiple comparisons, but * symbols give Bonferroni-corrected significance (* = $p < 0.05$, ** = $p < 0.01$, *** = $p < 0.001$).

Sector	w_a	w_g	w_s
Business	0.352	0.298	0.350
Scientific	0.422	0.305	0.273
Medical	0.428	0.297	0.275
Service	0.472	0.294	0.234
Industrial	0.441	0.328	0.231

Table S2: **Optimal weighting of automatability, job growth, and similarity for transition recommendations.** Rows represent sectors' weighted averages of optimal job-level values, where contribution to the sector average is weighted by the job's automation risk.

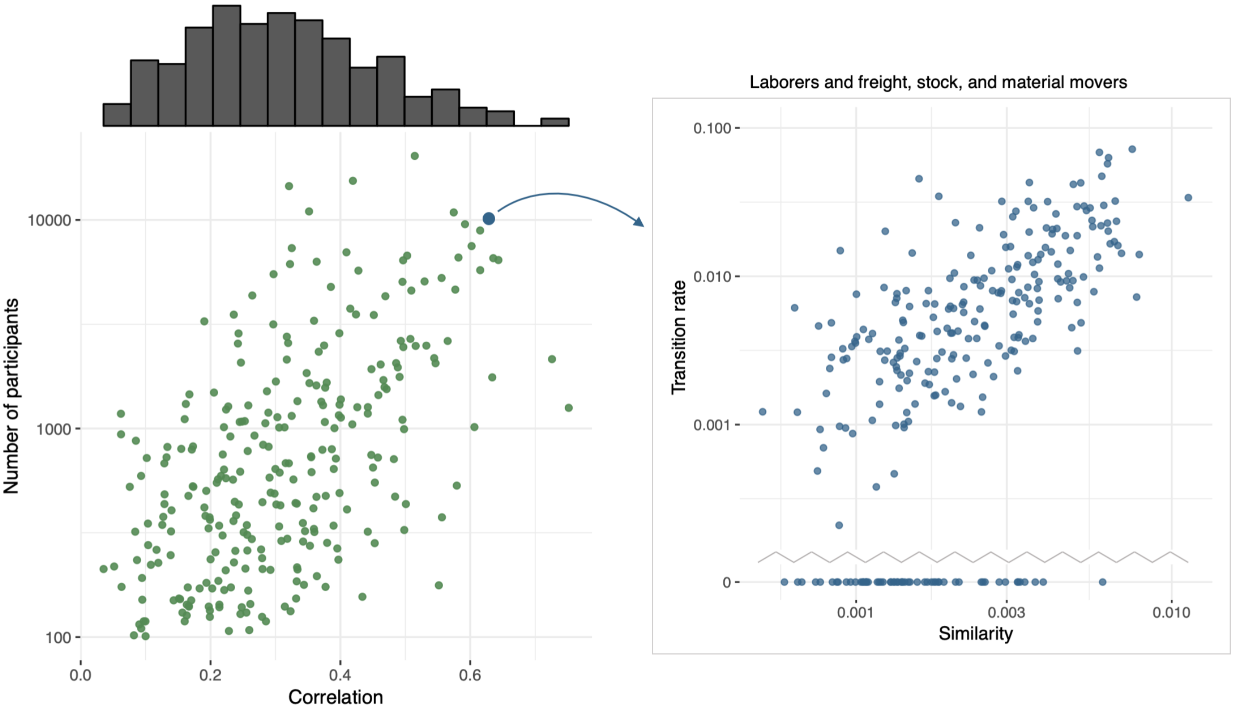

Figure S1: **Validation of pairwise job similarity measure**. Left panel shows the relationship between within-job similarity-transition correlations and the number of survey respondents for each job, as well as the overall distribution of within-job correlations. Right panel shows an example of the similarity-transition correlation for the job, *laborers and freight, stock, and material movers.*

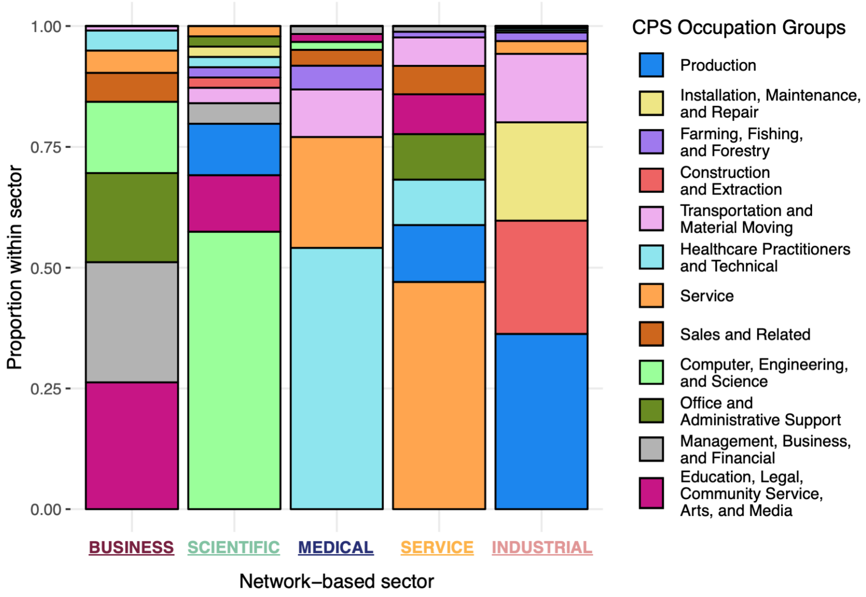

Figure S2: **Breakdown of CPS occupation groups within network-based job sectors**. Shows the proportion of jobs within each network-based job sector that come from one of twelve manually-assigned CPS occupation groups.

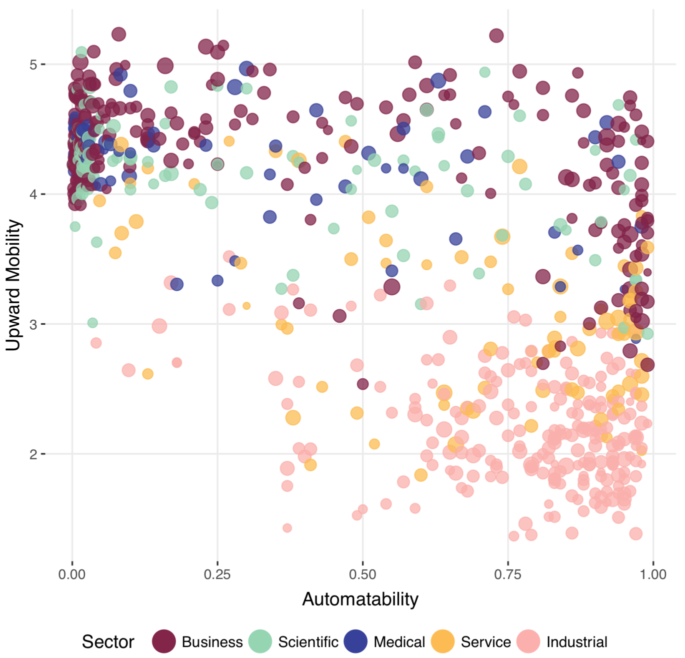

Figure S3: **Negative relationship between jobs' automatability and upward mobility**. Points represent individual jobs, which are colored based on sector. Figure displays negative association between automation risk and mobility to safe jobs, as well as differences in mobility by sector.

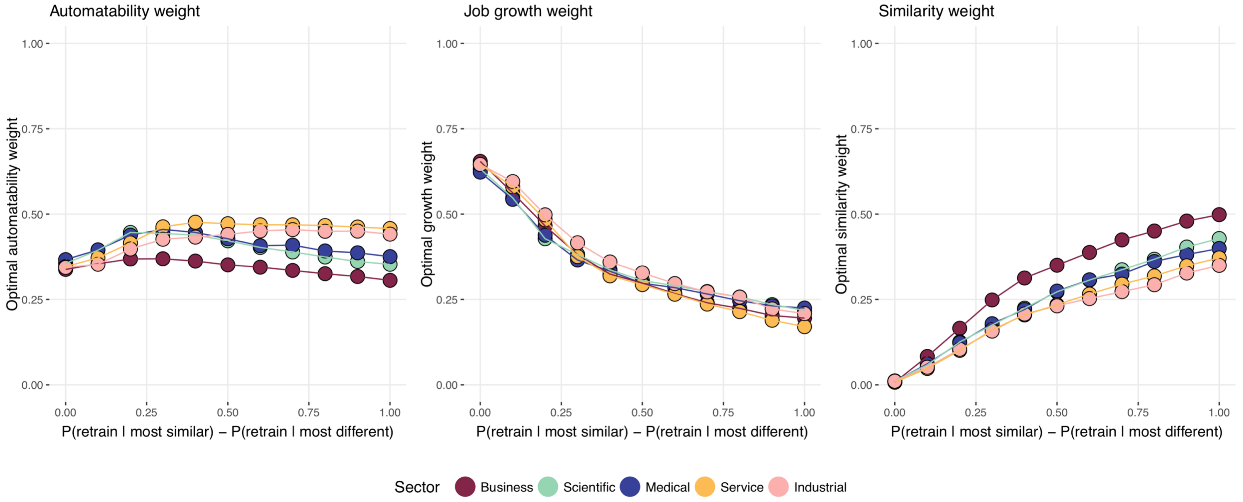

Figure S4: **Effect of translation from pairwise job similarity to retraining probability on optimal recommendation weights**. X-axes give the probability difference between retraining for the most similar job and the least similar job from a given starting point. From left to right, plots show the effect on optimal automatability, growth, and similarity weights. Values represent the average optimal weights for jobs within each sector, weighted by jobs' automation risk.

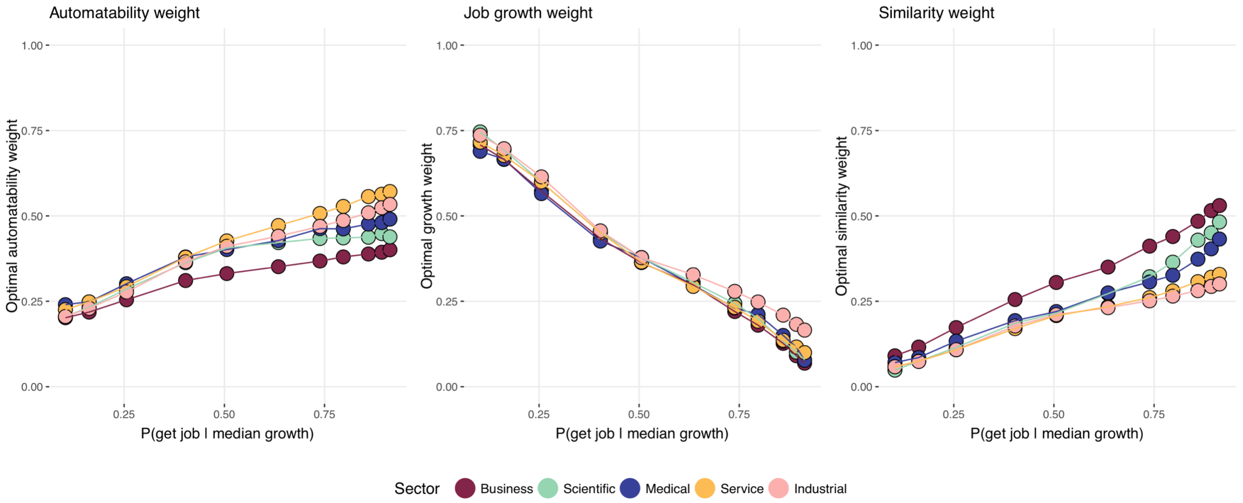

Figure S5: **Effect of translation from job growth to hiring probability on optimal recommendation weights**. X-axes give the probability of successfully obtaining a job with the median level of positive projected job growth, where the probability is always 1 for the job with the most growth. From left to right, plots show the effect on optimal automatability, growth, and similarity weights. Values represent the average optimal weights for jobs within each sector, weighted by jobs' automation risk.

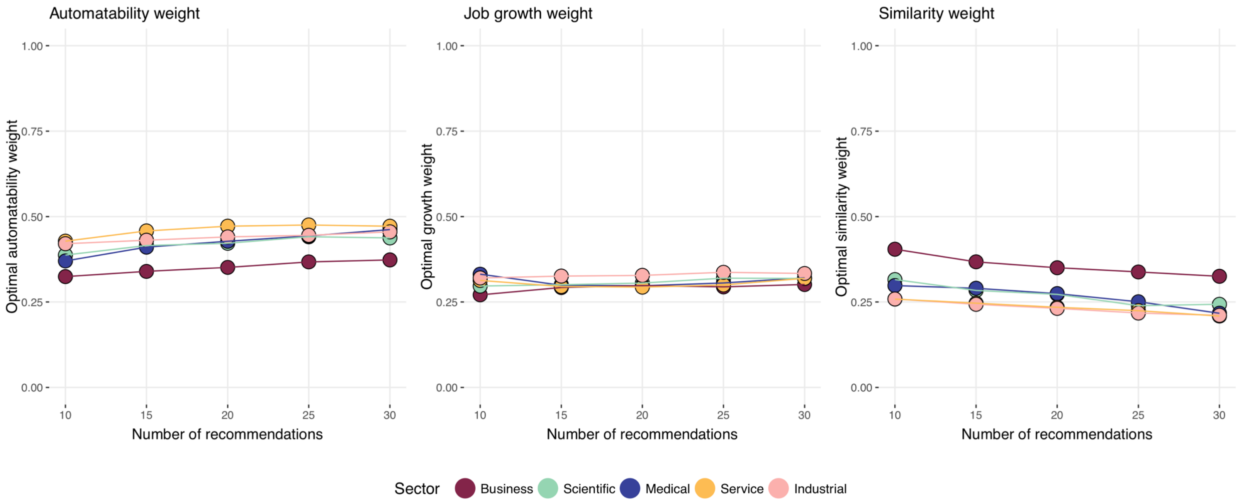

Figure S6: **Effect of the number of recommended jobs on optimal recommendation weights**. X-axes give the number of recommendations. From left to right, plots show the effect on optimal automatability, growth, and similarity weights. Values represent the average optimal weights for jobs within each sector, weighted by jobs' automation risk.

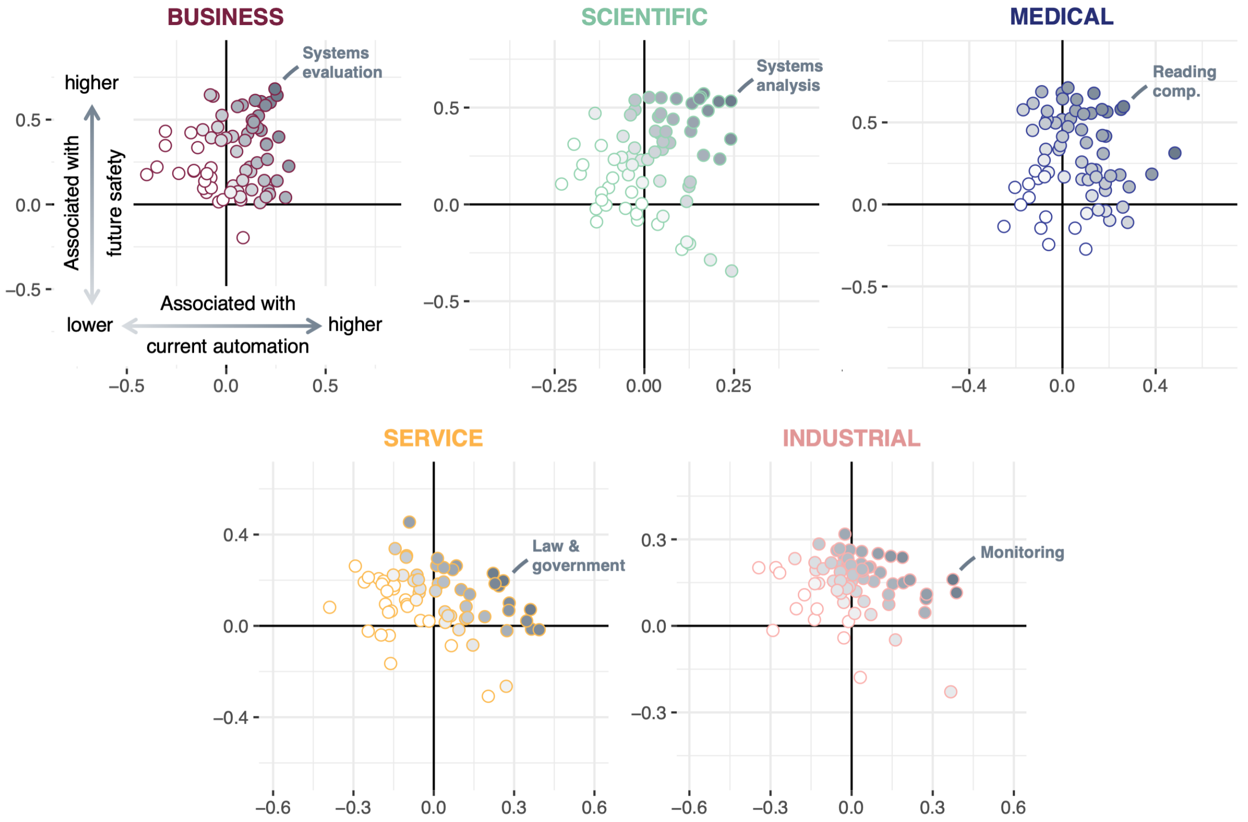

Figure S7: **Relationship between skills' associations with current automation and their associations with safety from future automation**. Panels show the association within each sector, where skills in the top right quadrant of each plot represent skills that are associated with both higher current automation and lower future automatability.

References

[1] Flood, S., King, M., Rodgers, R., Ruggles, S., Warren, J.R.: Integrated Public Use Microdata Series, Current Population Survey: Version 6.0 [dataset]. Minneapolis, MN: IPUMS (2018). doi: 10.18128/D030.V6.0. Accessed 2018-06-01

[2] Nelder, J.A., Mead, R.: A simplex method for function minimization. *The Computer Journal*, 7(4):308–313 (1965). doi: 10.1093/comjnl/7.4.308. Accessed 2018-07-16
